# Dynamic Adaption of Vascular Morphology

**DOI:** 10.3389/fphys.2012.00390

**Published:** 2012-10-02

**Authors:** Fridolin Okkels, Jens Christian Brings Jacobsen

**Affiliations:** ^1^Department of Micro- and Nanotechnology, Technical University of DenmarkLyngby, Denmark; ^2^Department of Biomedical Sciences, University of CopenhagenCopenhagen, Denmark

**Keywords:** vascular, morphology, adaptation, remodeling

## Abstract

The structure of vascular networks adapts continuously to meet changes in demand of the surrounding tissue. Most of the known vascular adaptation mechanisms are based on local reactions to local stimuli such as pressure and flow, which in turn reflects influence from the surrounding tissue. Here we present a simple two-dimensional model in which, as an alternative approach, the tissue is modeled as a porous medium with intervening sharply defined flow channels. Based on simple, physiologically realistic assumptions, flow-channel structure adapts so as to reach a configuration in which all parts of the tissue are supplied. A set of model parameters uniquely determine the model dynamics, and we have identified the region of the best-performing model parameters (a global optimum). This region is surrounded in parameter space by less optimal model parameter values, and this separation is characterized by steep gradients in the related fitness landscape. Hence it appears that the optimal set of parameters tends to localize close to critical transition zones. Consequently, while the optimal solution is stable for modest parameter perturbations, larger perturbations may cause a profound and permanent shift in systems characteristics. We suggest that the system is driven toward a critical state as a consequence of the ongoing parameter optimization, mimicking an evolutionary pressure on the system.

## Introduction

The arteriolar and venular trees supplying virtually all tissues in the mammalian body have long been recognized for their apparently regular, dichotomous branching pattern. Over a range of length scales these structures display a certain degree of self-similarity and can hence be characterized as quasi-fractal (Lorthois and Cassot, 2010). This regularity has led to attempts to define universal properties regarding vascular dimensions and network structure (West et al., 1997; Kassab, 2006). A famous example are the laws suggested by C.D. Murray almost a century ago (Murray, 1926a,b). These laws relate the radius of the mother vessel to those of the two daughter vessels. They also describe the branching angles when daughter vessels of equal or unequal size are formed from a mother vessel. Attempts to formulate in mathematical terms the laws (including those of Murray) that underlie vascular network structure, often take offset in considerations regarding energy conservation. It is assumed that network structure is optimized in such a way that the total energy requirement of the individual vessel (Taber, 1998) and the vascular tree as whole is minimized (Zhou et al., 1999; Liu and Kassab, 2007). This includes both the metabolic requirement of blood- and vessel wall components and the viscous energy dissipation of circulating the blood (Taber, 1998; Huo and Kassab, 2012). Not surprisingly, simulation studies applying energy optimization to guide network formation indeed lead to network structures similar to those observed experimentally (Karch et al., 1999). The principle of energy optimization combined with a few assumptions regarding the basic structure of the vascular system, has lead to the formulation of a number of scaling laws of the circulatory system that fit well to available data (West et al., 1997; Kassab, 2006; Huo and Kassab, 2012). It remains a central question however, by which mechanisms nature causes the realization of an energetically optimal structure at all levels from individual vessel to large networks.

The vascular wall is a highly plastic structure (Martinez-Lemus et al., 2009) that adapt dynamically to changes in local hemodynamic conditions e.g., pressure and flow (Unthank et al., 1996; Pourageaud and De Mey, 1997; Buus et al., 2001; Gruionu et al., 2012; Jacobsen and Holstein-Rathlou, 2012), which in many cases reflects a changing need of the surrounding tissue. A number of models have appeared in the literature taking this primarily “local” view on vascular adaptation (Hacking et al., 1996; Pries et al., 1998, 2001, 2003; Jacobsen et al., 2003, 2008; Nguyen et al., 2006; for a brief review please see Jacobsen et al., 2009). This plasticity indicates that the morphology of the individual vessel, and of the topology of the microvascular network as a whole, might result primarily from local adaptation to local conditions, rather than being a result of predetermined genetic patterning (le et al., 2005; Al-Kilani et al., 2008).

In the present model we aim at elucidating the hypothesis that changes in microvascular structure can be explained primarily as a local reaction to local conditions. As a novel approach tissue is modeled as an isotropic, porous material inside which size and position of flow channels are free to change. We formulated a set of generic equations describing vascular adaptation, and implemented them into a time-evolving numerical model applying the basic Navier–Stokes equation. In comparison to previous models of larger elastic arteries (Perktold et al., 1994; Formaggia et al., 2001), the present model is concerned with micro-vessels in which the pulse-wave is practically absent due to the damping effect of the upstream vascular tree. Hence the pressure decline across the model is considered to be constant. The model adapts morphology by defining structures (i.e., tissue regions and flow channels) on the basis of local cell densities the latter of which can vary in time. It includes supply and distribution of a general nutritive substance consumed in the tissue at a rate depending on cell density. This “general” substance is transported through the system by convection corresponding to blood flow in the living organism. On a smaller length scale it is transported by diffusion. We further introduced a general signaling substance as a messenger of local conditions. This signaling substance is created in response to critically low concentration of the nutritive substance and causes adaptation of the flow channels so as to regain a more uniform distribution of the nutritive substance in the tissue. As *in vivo*, flow channels are affected by shear (Langille and O’Donnell, 1986; Unthank et al., 1996; Matlung et al., 2009). This is mimicked in the model where, in the absence of sufficient shear, cells at the edge of flow channels expand spontaneously into the channel lumen.

The set of generic equations representing known adaptation mechanisms are composed of different terms. While these terms are fixed, their related control coefficients influence the adaption process, and initially this set of coefficients are globally optimized to mimic the presence of a long-term evolutionary pressure on the organism. Subsequently, with control coefficients remaining fixed during the short-term processes, the system is allowed to adapt dynamically to an imposed disturbance.

To test the adaption dynamics, a very simple model, consisting of only two flow channels through a tissue region, is forced into a highly un-favorable configuration, where the supply of nutrient to a large fraction of the cells is insufficient. This is done by deliberately closing one of the flow channels. Subsequently the model should be able to rearrange the structure so as to regain a more even distribution of nutrient, and suppress the formation of the signaling substance throughout the tissue.

## The Model

A central aim is to formulate the simplest possible two-dimensional (2D) model while retaining central features characterizing the microcirculation *in vivo*. Overall, the model consists of a set of governing equations; in the following each equation will be described, with emphasis on its relation to the known mechanics governing vascular adaption.

### Introduction of the model variables

In the following “*morphology*” is identical to the structure of the whole model system consisting of tissue regions (i.e., cells) and flow channels (i.e., vessels).

These elements introduce the following model variables: a scalar field-variable *c*, which is related to the distribution of cells, since it can be imagined as the local mean volume-fraction of cells at a given position. Consequently the upper value of *c* is limited by the value 1. The concept behind *c* will be discussed further in next subsection. The blood flow field is characterized by the 2D velocity *x*, *y*-components *u*, *v*, and the corresponding pressure *p*. The local concentration of nutrient is described by the scalar field-variable *a*, which is convected through the channels by the blood flow, and from here diffuses into the tissue. To further normalize the variables in the model, the inlet concentration *a*_0_ of *a* is set to the value 1.

For the model to dynamically optimize its morphology, it needs the presence of a signaling substance to distribute the information about local “shortage-of-nutrient” to the surroundings, and this is implemented as a scalar conc.-field *m*. By construction, the upper value of *m* is limited by the value 1. Similar to *a*, *m* is transported by diffusion within the tissue and primarily by convection inside the flow channels.

### Blood flow

In order to keep the model simple, non-Newtonian properties of the blood have been ignored. Consequently blood was assumed to behave as a simple, incompressible Newtonian fluid with constant density ρ and viscosity η. Blood is forced through the flow-channel(s) by a constant pressure gradient mimicking the decline in pressure along the microvascular bed *in vivo*. Inside the flow channels mass-transport is dominated by convection.

To model the damping of the flow as it passes through the tissue capillary network, we imagine tissue regions (all regions outside flow channels) as consisting of a porous material with porosity imitating capillary density. Hence in regions where capillary density is high, porosity is high, and *vice versa*. Porosity is therefore inversely related to the volume-fraction of tissue-cells (i.e., both parenchyma and stroma) in a given region. This property is implemented in the governing flow equations by introducing a Darcy damping force, i.e., a volume force, where each fluid molecule is subjected to a force, proportional to the flow-velocity, but acting in opposite direction of the flow so as to damp the motion. The coefficient of this damping force, α, can be viewed as the inverse of local permeability, i.e., low values enhance the ability of the flow to penetrate the tissue and *vice versa*. In the tissue regions mass-transport is dominated by diffusion.

Collectively, the governing equations for the flow become the basic Navier–Stokes equation with the additional Darcy damping term (Eq. 1) together with the continuity equation for incompressible fluids (Eq. 2).

$$\begin{array}{llll} & \rho \left(\frac{\partial \vec{u}}{\partial t}+\left[\vec{u}\cdot \nabla \vec{u}\right]\right)=-\nabla p+\eta \nabla^{2}\vec{u}-\alpha \;\left(\vec{r}\right)\;\vec{u}, & & \text{(1)}\\ & \nabla \cdot \vec{u}=0, & & \text{(2)} \end{array}$$

where ρ and η are fluid density and viscosity, respectively, and where $\rho \left(\frac{\partial \vec{u}}{\partial t}+\left[\vec{u}\cdot \nabla \vec{u}\right]\right)$ is the inertial term, −▽*p* is the pressure driving term, $\eta \nabla^{2}\vec{u}$ is the viscous dissipation term, and where $-\alpha \left(\vec{r}\right)\vec{u}$ is the Darcy damping term. The damping coefficient is $\alpha \left(\vec{r}\right)=-\alpha_{\max}c$, with $\alpha_{\max}$ being the maximal damping coefficient, i.e., in regions where the tissue is maximally dense.

### Description of model layout

**Figure 1:**
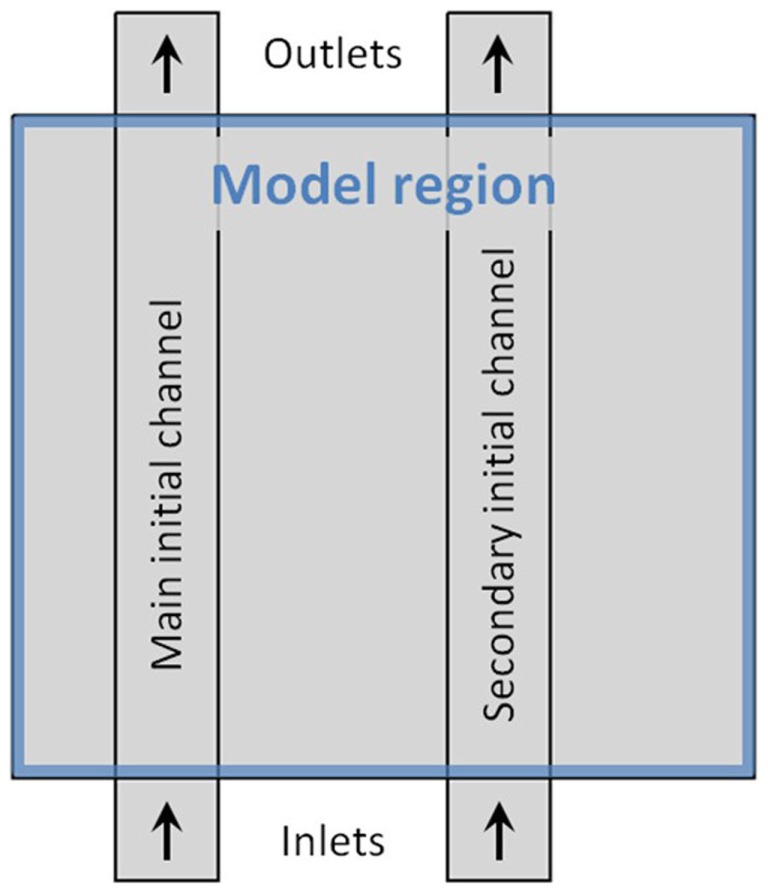
**Schematic illustration of the model layout**. The blue frame marks the model region, where the dynamics takes place. Inlet- and outlet-areas are marked by arrows. Vertical rectangles show the main and secondary channel-configuration, used to initiate the dynamic adaptation of the model. To set the overall size of the model, each initial channel is 4 μm wide, and the model region measures 29 × 26 μm.

The model region, within which morphology is free to change during the dynamic adaptation process, is confined within a rectangular area, as illustrated in Figure 1. All exchange of blood with the surroundings take place between one or two inlets at the lower boundary of the model region and one or two outlets at the upper boundary. The blood is driven through the model tissue by a constant pressure difference across the tissue mimicking a fraction of the pressure gradient generated by the heart in the mammalian vascular system. As shown in Figure 1 inlet and outlet regions for the flow channels extend beyond the model area. This is done to ensure that as fluid enters or leaves the model region, effects relating to the model boundary itself is minimized. The pressure difference across the system is only applied at the in- and outlet boundaries; all remaining boundaries in the model are hard walls i.e., non-permeable to blood or any other substance. To set the overall size of the model, each initial channel is set to a width of 4 μm, with an overall dimension of the rectangular model region being 29 μm × 26 μm.

### Species transport

At any given time, morphology (i.e., tissue density and position of flow channels) is the sole determinant of how blood flows through the system. This happens through the action of the Darcy damping term (Eq. 1) that efficiently damps any flow in the tissue regions. Furthermore to reduce the complexity of the model, we do not distinguish between interstitial fluid and blood. Once the blood flow is known, it affects the general transport equations, as shown in Eq. 3, which take into account both mass convection and diffusion.

(3) $$\frac{\partial s}{\partial t}+\vec{u}\cdot \nabla s=D_{s}\nabla^{2}s+R_{s},$$

By replacing the general scalar *s* with one of the three concentration-fields of the model: nutrient concentration *a*, the concentration of signaling substance *m*, or the local mean cell density *c*, the equation describes how the different species spread through the model.

However, since cells have a fixed position in the tissue, the convection term, $\vec{u}\cdot \nabla c,$ vanishes for the local cell density *c*. Still cells are able to move slightly, mimicking cellular migration *in vivo*, as expressed through the diffusive term, $D_{s}\nabla^{2}c$.

### Dynamics arising from the coupling terms *R_s_*

The actual system dynamics arise from the combined source/drain terms *R_s_* and their mutual couplings:

#### The nutrient uptake rate: *R_a_*

The uptake rate of nutrients is chosen as simple as possible, that is, making the uptake proportional to: the local cell density, a nutrient uptake factor, *M_f_(a)*, and to an overall uptake rate constant *k_a_*, collectively giving:

(4) $$R_{a}=-k_{a}\;c\;M_{f}\left(a\right).$$

*M_f_(a)* plays an important role, both directly in the uptake rate of the nutrient and as a critical parameter controlling the release of the substance signaling insufficient flow. *M_f_(a)* is defined as a normalized version of the well known Hill-type expression (Murray, 1993) for enzyme kinetics:

(5) $$M_{f}\left(a\right)\equiv \frac{a^{n}}{K+a^{n}},$$

where *K* is the Hill constant, and where the Hill exponent *n*, equals 2 throughout. To better interpret the action of the Eq. 8 in a later discussion of the model dynamics, we introduce a characteristic “Hill-concentration” which is $a_{H}=\sqrt[{n}]{K}$, and which divides the saturation at high concentration (*a* > > *a*_H_) from the power-law dependence at low concentration (*a* < < *a*_H_).

#### Temporal change in cell density: *R_c_*

The signaling substance *m*, is generated by metabolically stressed cells, that is, cells exposed to insufficient concentration of *a*. The overall effect of *m* is to diffuse out into the surroundings and cause alteration of tissue morphology so as to ultimately achieve an appropriate level of perfusion. We can decompose the direct rate-of-change term *R*_c_ of the local cell density into two terms:

A *cell growth* term, *k*_growth_*c*^2^(1 − *c*), which only depends on the local cell density in a non-linear fashion, and whose effect is to let cells grow continuously, both within tissue regions reaching toward *c* = *1* and into the channel lumen, if cell growth is not inhibited by the second term:

A *shear rate* term, $k_{\text{Shear}}c\;m\;\dot{\gamma }$, whose primary function is to open the tissue for transport of nutrients from nearby flow channels. This is done, as shown in Eq. 6, by incorporating *m* into the rate equation controlling cell density and thereby making it active only in situations where the signaling substance is released nearby.

Second, due to the mean field approach of the model, i.e., that the micro-structure capillary network is modeled as a homogeneous porous material, shear stress arise only on the larger channel walls, and we can therefore limit the action of the shear term by introducing a rough estimate of the shear stress $c\cdot \dot{\gamma },$ with the local 2D shear-flow rate $\dot{\gamma }$. With appropriate rate coefficients we arrive at the following expression:

$$R_{c}=k_{\text{growth}}\;c^{2}(1-c)-k_{\text{shear}}\;c\;m\;\dot{\gamma },$$  (6)

where the shear-flow rate $\dot{\gamma }$ only depends on the local flow characteristics:

(7) $$\dot{\gamma }=\sqrt{2{\left(\frac{\partial u_{x}}{\partial x}\right)}^{2}+{\left(\frac{\partial u_{x}}{\partial y}+\frac{\partial u_{y}}{\partial x}\right)}^{2}+2{\left(\frac{\partial u_{y}}{\partial y}\right)}^{2}}.$$

#### Release- and decay-rate of the signaling molecule: *R_m_*

Since the main purpose of the signaling substance is to respond to insufficient perfusion, in this case signaled by an insufficient concentration of the nutrient *a*, we introduce the following *Stress factor*, $1-c\;M_{f}\left(a\right)$, the main determinant of the local production of *m*. This factor is given by the difference between the ideal uptake rate that is close to unity [as *M_f_*(*a*_0_) ≈ 1], and the actual uptake *cM*_f_(a).

The next factor, 1−*m*(2−*c*)^β^, is denoted the *Scavenger factor*, which acts to remove *m* as it reaches the channel lumen. Hereby it dynamically relates *m* and *c* in order to obtain sharply defined channel walls. Here the exponent β, which is larger than 1, sets the *m*-*c* relation.

Combining these factors with an overall rate factor *k_m_* *c*, restricting the effect to tissue regions, the rate-of-change of the signaling substance is given by:

(8) $$R_{m}=k_{m}\;c\;\left[1-c\;M_{f}\left(a\right)\right]\;\left[1-m{\left(2-c\right)}^{\beta }\right].\;$$

**Figure 2:**
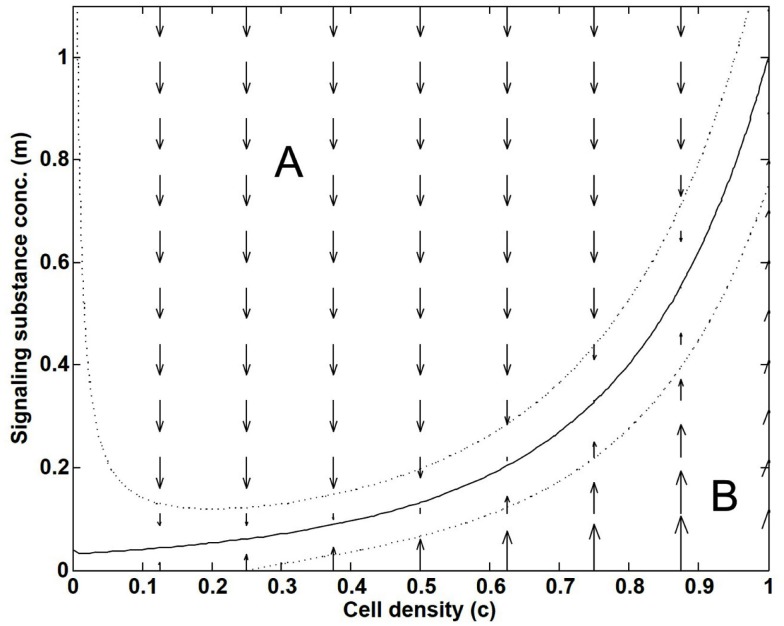
**Illustration of how the “Scavenger factor” in Eq. 8 dynamically creates a local relation between the variables *c* and *m***. As a function of cell density *c* and the signaling substance concentration *m*, the response from the “Scavenger factor” on the rate-of-change R_m_, and thereby on the dynamics of *m*, is shown by arrows. *m* and *c* will asymptotically be driven toward the solid black zero-line in the final steady-state. The two dotted lines correspond to contours of the size of the Scavenger factor equaling ±1/2.

Figure 2 shows the action of the Scavenger factor, where convection and diffusion have been neglected i.e., studying the simplified dynamics $\frac{dm}{dt}=R_{m}$, and where we assume *m* to be active, that is, non-zero. Keeping *c* fixed, the arrows show the direction of change in *m* that comes from the Scavenger factor alone. In the region A of Figure 2, the value of *m* will decrease, while it will increase in the region B. As a result, *m* and *c* will asymptotically be driven toward the solid black zero-line at a final steady-state. The two other dotted lines correspond to contours of the size of the Scavenger factor equaling ±1/2.

In order for the shear term in *R*_c_ to balance the growth term, a small concentration of *m* has to be present throughout the modeling region at all times, and this is ensured both by feeding the model with a small inlet concentration of m (denoted m_0_) and by the always non-zero generation of *m* from the “Stress factor” term in *R*_m_.

### The numerical implementation

#### Numerical method

We have implemented the model into the commercial software package COMSOL, where we solve the full dynamic adaption process by a sequential use of both steady-state and time-dependent partial-differential-equation solvers. This sequence is described in more details in the section “Model evaluation”.

#### Consequences of solving the model numerically

It is important to take into consideration the method used for solving numerically the time-evolution of the model (i.e., the integration of the model). In general, an exact mathematical solution can only be found for simple models, whereas solving more complex systems requires the use of approximate numerical methods. Numerical integration methods are inevitably associated with small errors, which may accumulate or in other ways influence and perturb the resulting solution. Hence, consideration should be given to the potential consequences of the numerical method itself.

In the present case when attempting to solve numerically the system of equations presented earlier in this section, it is soon realized that under certain conditions the solution may become un-physical, with e.g., concentrations becoming negative, which may in turn lead to false reactions-term values etc. in the model.

A simple mathematical analysis of the dynamical model system reveals that such flaws should not be possible, however, since the numerical method introduces small errors to the field-variables, a very low value of e.g., the concentration of a given species, can result in a “jump” into a negative region. If the reaction by the model to such un-physical values is to further enhance an incorrect tendency, the model finally produces overall un-physical behaviors^1^ (Strogatz, 1994).

In the following we show which measures are taken to prevent such flaws.

#### The nutrient uptake factor: *M_f_*(a)

The nutrient uptake factor, *M_f_(a)*, should in principle never be evaluated for negative nutrient concentrations, but should this happen, the original expression would result in a positive valued *M_f_(a)* that would drive *a* further downward. To prevent this, in the actual implementation *M_f_(a)* is split up, as shown below, such that for negative values of *a*, it returns the value of *a* itself, thus stabilizing the dynamics:

(9) $$\begin{array}{l} M_{f}(a)\equiv \frac{a^{n}}{K+a^{n}},\;a\geq 0\\ M_{f}(a)\equiv a,\;a<0. \end{array}$$

#### Temporal change in cell density: *R_c_*

Similarly for the nutrient uptake factor, the temporal change in cell density *R*_c_ may be caught by negative values of the cell density *c*. As for *M_f_(a)*, in the actual implementation *R*_c_ is therefore split up, depending on the sign of the cell density *c*, thus altering the response from the “cell growth” term, while keeping the “shear response” term fixed:

(10) $$\begin{array}{l} R_{c}=k_{\text{growth}}\;c^{2}(1-c)-k_{\text{shear}}\;c\;m\;\dot{\gamma },\;c\geq 0,\\ R_{c}=-c-k_{\text{shear}}\;c\;m\;\dot{\gamma },\;c<0, \end{array}$$

### Evaluation of the dynamics

The dynamics of the model is constructed to be transient, such that the temporal evolution of the model variables comes to rest in a steady-state within a limited timeframe. This makes it possible to define a final state of the dynamics, characterized by a specific channel morphology. This final channel morphology is highly dependent on the given choice of model parameters (i.e., *k*_a_, *m*_0_, β, *K*_growth_, *K*_shear_, and *K*), which controls the influence of the different coupling terms on the model dynamics. In that way, each specific set of model parameters (fixed while solving the model dynamics) uniquely controls the morphology of the final state, since the model is always initiated from the same un-favorable channel morphology.

As already mentioned, the best choice of model parameters are found through an optimization procedure, mimicking a long-term evolutionary pressure to maximize the fitness of the whole biological entity in question. This optimization method is not linked to the actual model dynamics, since it is based only on an evaluation of the final steady-state performance.

The general way of determining performance within optimization theory is to define an objective function Φ(**u**, **γ**), which evaluates all the variables of the model, denoted by **u**, for a given set of model parameters, denoted by **γ**, and returns a single scalar number, which by convention has to be minimized during the optimization procedure. To ensure that the model has sufficient time to reach a final state, regardless of the configuration of the model parameters, we terminate the temporal solution of the model at *t*_∞_ = 1331/3, which has been chosen two orders of magnitude larger than the average transition time of the model dynamics, and we can therefore specify the objective function further as Φ[**u**(t_∞_),**γ**].

Based on the overall aim of the model to evenly distribute nutrition in the tissue, a natural choice of objective function would be to measure the average metabolic uptake rate *R*_a_ within the model region, Ω, and thus use the objective function:

(11) $$\Phi_{R_{a}}\left[\text{u}\left(t_{\infty }\right),\gamma \right]=\frac{1}{k_{a}}{\left\langle R_{a}\left(t_{\infty }\right)\right\rangle }_{\Omega }=\frac{1}{k_{a}\left|\Omega \right|}\int_{\Omega }R_{a}\left(t_{\infty }\right)\text{d}\vec{r},$$

where the brackets ${\left\langle \ldots \right\rangle }_{\Omega }$ denote an averaging over Ω.

As described in the following, the objective function Φ_Ra_ alone does not guide the optimization procedure toward the best-performing state. Therefore we introduce an additional objective function based on the presence of the signaling substance, where we aim at minimizing the average squared concentration of signaling substance:

(12) $$\Phi_{m}\left[\text{u}\left(t_{\infty }\right),\gamma \right]={\left\langle m^{2}\left(t_{\infty }\right)\right\rangle }_{\Omega }=\frac{1}{\left|\Omega \right|}\int_{\Omega }m^{2}\left(t_{\infty }\right)\text{d}\vec{r}.$$

It should be stressed that these objective functions only evaluate the performance of the final state of the whole model i.e., how well the model, at *t* = *t*_∞_, did recover from the initial un-favorable channel morphology. This is reflected in the objective functions, which measure mean properties throughout the whole model region. *In vivo*, all tissues, both developing and mature, have the potential to optimize vascular structure. Hence, although the use of the objective functions evaluating this ability are here limited to a small model region, they in principle apply to the complete organism. This should not be confused by the fact that the vascular adaptation during model dynamics is only based on local stimuli, i.e., the short-range transport of the signaling substance *m*.

The overall optimization procedure, i.e., finding model parameters that results in the best-performing final channel morphology, consists of first using Φ_Ra_ until the global optimum in the model parameter space is found^2^. At this configuration the related model dynamics show signs of vascular adaption, but as Φ_Ra_ favors large tissue regions at the expense of sufficiently supplying these regions, we consider this choice of model parameters as an intermediate step toward the best performance of the model. Therefore, we then continue the optimization using Φ_m_ toward a final optimized set of model parameters, which are presented in the Result section. Now that the optimization is guided by Φ_m_ it ensures that the tissue becomes adequately supplied by nutrition. The reason why Φ_m_ cannot be used exclusively throughout the optimization procedure is that Φ_m_ has the trivial but global optimum when all tissue is removed from the model region.

## Results

### Model parameters

Even though the present model is best described as a conceptual model of dynamic vascular adaption, some of the physical model parameters, which are presented in Table 1, have values that correspond to realistic values when given in SI-units, e.g., the density and viscosity of water. Furthermore, the following realistic model parameter values have been chosen: Inlet/outlet channel widths (4 μm), the diffusion coefficient of the nutrient (1.5 × 10^−9^ m^2^/s)^3^, and the average blood flow-velocity (∼1.5 mm/s (Zweifach and Lipowsky, 1977) resulting from the choice of a fixed pressure drop across the model.

**Table 1:** **Model parameters for the best-performing model state, with an identification of whether the parameters are fixed or allowed to vary during the optimization procedure**.

Δp	75	Fixed
ρ	10^3^	Fixed
η	10^−3^	Fixed
α_max_	2.5 × 10^10^	Fixed
k_a_	37.5	Fixed
m_0_	1.2 × 10^−3^	Fixed
k_m_	71.14	Optimized
β	5.2	Optimized
K_growth_	322.7	Optimized
K_shear_	3.3	Optimized
K	10^−2^	Fixed
D_a_	1.5 × 10^−9^	Fixed
D_c_	3 × 10^−11^	Fixed
D_m_	6 × 10^−10^	Fixed

In order to keep the computational load manageable the model region spans only 29 μm × 26 μm. For the imposed feedback mechanisms that causes adaption to be realizable in such a limited region, the different reaction rates in the model is scaled up, i.e., all adaptive responses in the model occurs within 10 time units. Since we are investigating a conceptual model of dynamic vascular adaption, the time-scale of the model has no real meaning, and it therefore only serves to identify the chronology of the dynamics.

Special attention has been paid to the three parameters *k*_a_, *m*_0_, and *K*, which are fixed during optimization, as seen in Table 1, but which could equally well be seen as parameters free to change during optimization. They are fixed because their impact on the model dynamics has been unambiguously resolved during the analysis of the model response. Fixing these parameters at well-chosen values decrease the dimensionality of the optimization parameter space, and thereby dramatically reduce the computational time needed in searching for the final model parameters. To carry out most of the parameter optimization, we use a direct bounded Nelder–Mead simplex method (Nelder and Mead, 1965) implemented in Matlab (Lagarias et al., 1998).

### Model evaluation

As mentioned earlier, the way the model-generated adaption is evaluated, is to change the state of the model from a favorable state into a highly un-favorable state, and subsequently evaluate how the model “recovers.”

#### Initial steady-state with two channels

**Figure 3:**
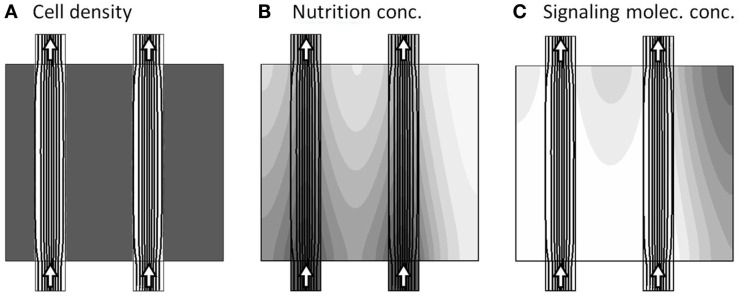
**Model variables at the pre-initial state, where both channels are active as seen by the arrows**. The three sub-figures **(A–C)**, show the distribution of c, a, and m, respectively, in gray-scale coded contour surfaces ranging from white (0) to dark gray (1), with black lines showing the stream-lines of the flow-field.

To set the pre-initial state, where both channels are active, and before the onset of dynamical changes, we fix the cell morphology of the model to consist of dense tissue, except from the two free channels, denoted the main and secondary channel (see Figure 1). Keeping this configuration, and finding the flow-field for this configuration, we further initiate this pre-initial state by obtaining the steady-state distribution of the nutrition and signaling substance field-variables *a* and *m*, i.e., solving the model system without the presence of the temporal derivatives: $\frac{\partial a}{\partial t}\equiv 0$ and $\frac{\partial m}{\partial t}\equiv 0$. The resulting model variables are presented in Figure 3, where the three sub-figures shows the distribution of *c*, *a*, and *m*, respectively, in gray-scale coded contour surfaces, ranging from white (0) to dark gray (1)^4^, with black lines showing the stream-lines of the flow-field^5^.

Two important properties are visible in the pre-initial state, as shown in Figure 3. First there is a steady “consumption” of nutrient by the tissue, as seen by the decrease in gray tone when going downstream along the channels from the inlets and transversally into the tissue (Figure 3B). Second, the signaling substance concentration is generally low, except from a small build-up in the upper right corner of the model region, caused by slightly poorer nutrition supply in that area (Figure 3C).

#### Dynamics from one channel

**Figure 4:**
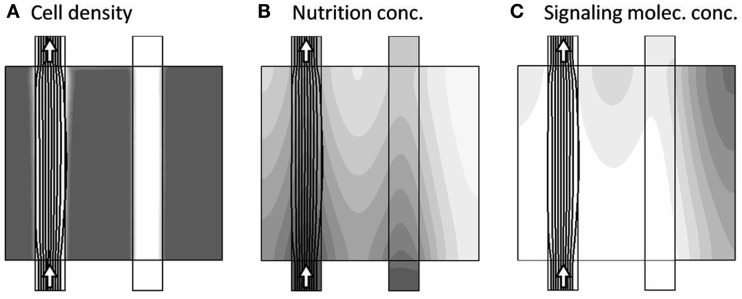
**Model variables, now at the onset of the dynamics (model time tA = 2.5 × 10^−4^), where only one channel is active as seen by the arrows**. The three sub-figures **(A–C)**, show the distribution of c, a, and m, respectively, in gray-scale coded contour surfaces ranging from white (0) to dark gray (1), with black lines showing the stream-lines of the flow-field. The only major change compared to Figure 3 is the blocking of the secondary channel, as the nutrient, supplied by the secondary channel, not yet has been completely consumed.

The actual dynamics begins by first closing the secondary channel to the right, and then allow all model variables: *u*,*v*,*p*,*c*,*a*, and *m* to vary in time according the dynamical equation-system. The general adaption dynamics consists of three phases, with the related time-scales *t*_A_, *t*_B_, and *t*_C_, as described in the following. Very early, corresponding to model time *t*_A_ ≈ 2.5 × 10^−4^, the only major change is the blocking of the secondary channel, as seen in Figure 4, as the nutrient, supplied by the secondary channel, has not yet been consumed.

**Figure 5:**
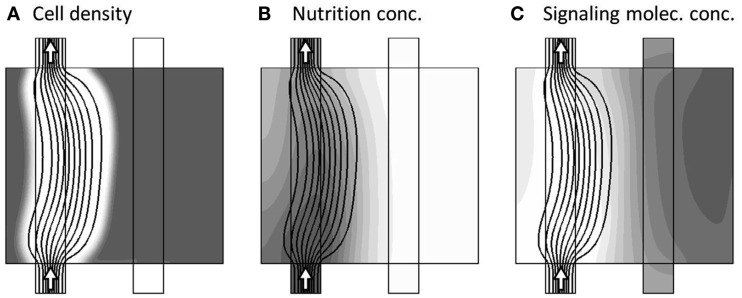
**Model variables, now at the incident of maximum signaling substance concentration (model time t_B_ = 0.075)**. The three sub-figures **(A–C)**, show the distribution of c, a, and m, respectively, in gray-scale coded contour surfaces ranging from white (0) to dark gray (1), with black lines showing the stream-lines of the flow-field. The morphology is already reacting to the presence of *m* by diverting the transport of nutrient into the “stressed” right part of the model region.

Once the nutrient in the right part of the model region has been consumed, the tissue reacts by releasing the signaling substance, and at model time *t*_B_ ≈ 0.075, where the concentration of the signaling substance reaches a maximum, the morphology is already reacting to the presence of the signaling substance, gradually changing such as to divert the transport of nutrient into the “stressed” right part of the model region. This is shown in Figure 5.

**Figure 6:**
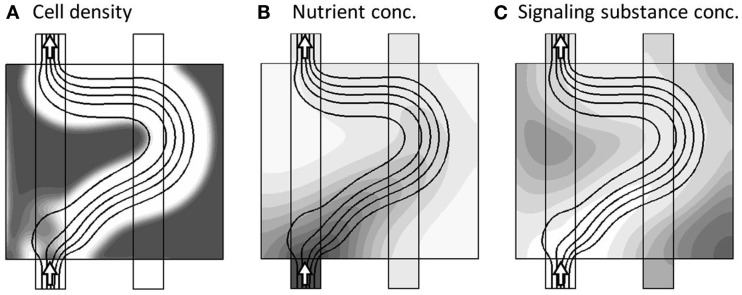
**Model variables, now at the final steady-state of the model dynamics** $\left(t_{c}\approx t_{\infty }=\frac{1331}{3}\right)$. The three sub-figures **(A–C)**, show the distribution of c, a, and m, respectively, in gray-scale coded contour surfaces ranging from white (0) to dark gray (1), with black lines showing the stream-lines of the flow-field. The final stage of adaption, where the different regions are now more evenly supplied.

This morphological adaption continues until all regions are again sufficiently supplied. In turn, this brings the concentration of signaling substance below the critical value, such that the dynamics comes to rest in a final steady-state, as shown in Figure 6.

### Morphological changes

**Figure 7:**
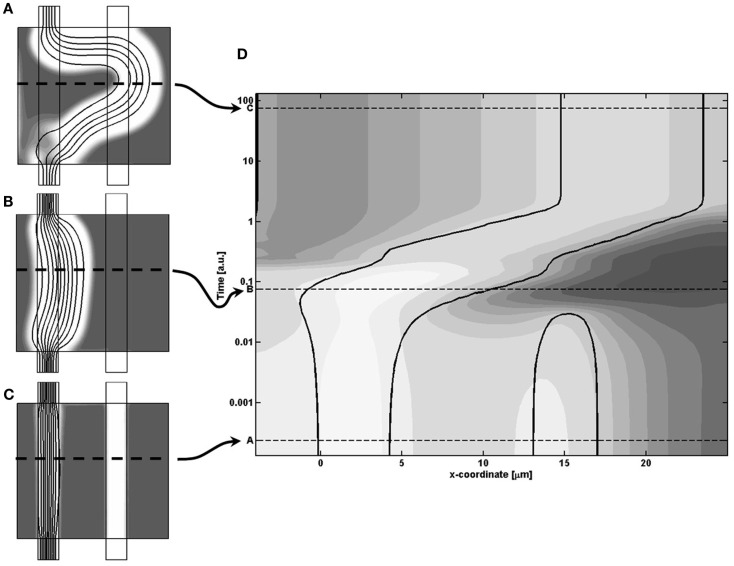
**(A–C)** Inserts of the cell distributions at different model times, related to **(D)** A spatio-temporal plot of the concentration of *m* given by gray-scale coded contour surfaces, and showing the continuous dynamical adaption of the model, sampled from the horizontal cut identified by the dashed line in the inserts. In **(D)** the morphological change of the cell density is illustrated by the solid black contour lines of cell density equaling the value 1/2.

To investigate the coupling between release of the signaling substance, and the morphological changes, we sampled the temporal variations of how the model field-variables changes along a horizontally positioned cut centrally in the model region. In that way we can let the vertical axis represent time, while the horizontal axis is the x-coordinate along the model region and thereby visualize the continuous change in model variables during the evolution of the system, as shown in Figure 7. Here the variations of *m* is given by gray-scale coded contour surfaces in the spatio-temporal plot (D), where the morphological change of the cell density is illustrated by the solid black contour lines of cell density equaling the value 1/2, and thereby representing how the channel boundaries change with time. The three instances, described earlier, are shown by the three dashed lines A–C in plot (D), with the sampling cut position shown by dashed lines in insets (A–C). To cover the large span in the different time-scales of the model dynamics, we let the vertical axis be the logarithm of the model time.

Going through the chronology of events shown in Figure 7 we first see the closing of the secondary channel to the right, followed by a build-up of the signaling molecule concentration. From then on, the figure illustrates the coupling between the critical presence of signaling molecule *m* and the resulting dynamic adaption of morphology, based on the following responses: the right side of the main channel wall broadens into the surrounding tissue since the high local concentration of *m* makes the shear rate term dominate over the cell growth term in the *R*_c_ expression (Eq. 6). On the other hand, the weak concentration of *m* let the cell growth term take over such that the left channel wall expand into the channel lumen, and thereby keep the channel width fixed. During the channel adaption, there is a weaker build-up of *m* on the left side, but these concentrations never become critical, i.e., the two terms retain their ratio, and therefore do not prohibit the expansion into the channel lumen.

### Robustness of the model

To ensure that the efficient adaptation dynamics is a robust feature of the model, and not just a fortunate coincidence for a single model realization, we have tested the model response to the following perturbations: All the optimized parameters (*k*_m_, β, *k*_growth_, *k*_shear_) have been varied, and the best-performing state, as seen in Figure 6, remained as final configuration within perturbations of ±5% from the values given in Table 1. Furthermore a ±5% variation of the parameter *K* and the Hill exponent *n* in Eq. 6 did not change the final configuration. To test the numerical convergence of the model at the best-performing state, we refined the mesh-size of the triangular meshing from the original 7 × 10^−7^ μm down to 2 × 10^−7^ μm without any spatio-temporal change of the model dynamics or in the final state.

### Criticality in the model

**Figure 8:**
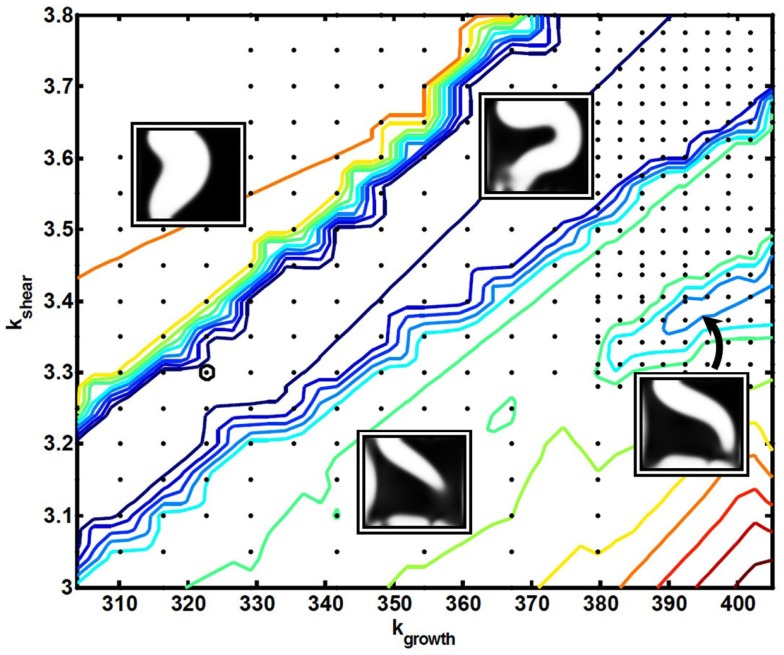
**A contour plot of the objective function Φ_m_ as a function of the model parameters k_growth_ and k_shear_, in the vicinity of the best-performing parameter-value (bold black circle)**. All evaluated parameters are represented by black dots, and color-coding of the contour lines span from best performance in dark blue to poorest performance in dark red. Four inserts have been placed at each of the three regions, plus one related to a local optimum. All inserts show the corresponding final cell density in gray-scale.

An underlying premise in this work is that the evolutionary pressure constantly adjusts the model parameters over long time-scales, such as to optimize the adaption dynamics. With this conceptual model, we are able to scan the model parameter space around the final set of parameters, and it turns out that the best-performing parameter-values lie close to abrupt transitions in adaption performance. Passing through such transitions, the corresponding final states of the adaption dynamics end up at poorly performing channel configurations. Figure 8 shows the parameter-plane spanned by *k*_growth_ and *k*_shear_ in the vicinity of the best-performing parameter-value (bold black circle). All evaluated parameters are represented by black dots, and colored contour lines show the values of the objective function Φ_m_, spanning from blue as the best-performing, lowest values up to the red, poorest performing, highest values. Clear sharp transitions are seen between the middle region of best performance and the two “failure”-regions on both sides. To illustrate the types of failure, four inserts, showing the corresponding final cell density in gray-scale, have been placed at each of the three regions plus at a small area to the right of Figure 8 (indicated by an arrow), corresponding to a local minimum of the objective function. Such local minima exist throughout the parameter space, which complicates the search for a global minimum.

**Figure 9:**
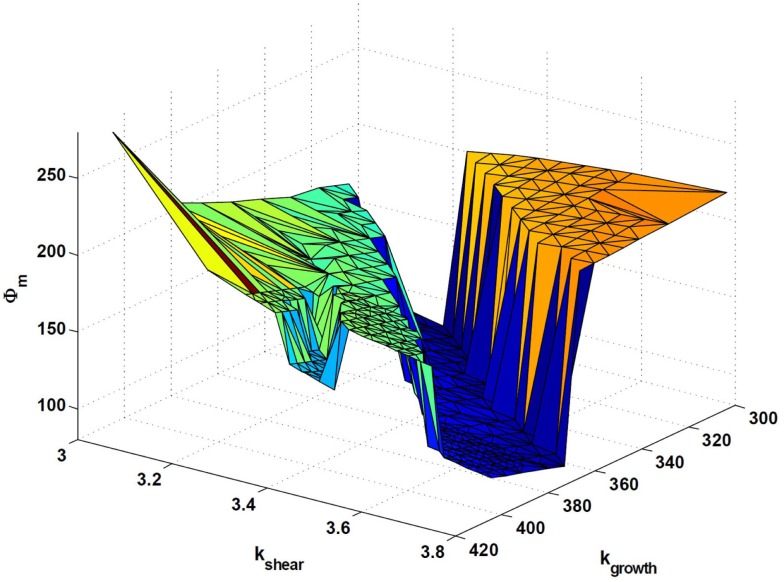
**Three-dimensional surface of the objective function Φ_m_ as a function of the model parameters k_growth_ and k_shear_, which is another visualization of the same data as presented in Figure 8**. A sharp transition is clearly seen between the central “valley”, containing the best-performing state, in the objective landscape, and the surrounding “cliffs”, marking the transitions to poorer performing states.

To further visualize these transitions, Figure 9 shows the three-dimensional (3D) objective “landscape”, which presents the same data as in Figure 8, but now with Φ_m_ on the vertical axis. A sharp transition is evident between the central “valley”, containing the best-performing state in the objective landscape, and the surrounding “cliffs”, marking the transitions to more poorly performing states. One should also note that the best-performing region tilts slightly toward the right in Figure 9. This means that the actual optimal parameter-value lies on the transition, and therefore that a pure optimization process of inconsiderately moving toward better performance in parameters space would lead to spontaneous failures. Although it remains speculative whether it also applies to real-world systems, a consequence emerging from the present conceptual model is that criticality may arise naturally from the action of an evolutionary pressure.

Due to the relative simplicity of the dynamic model, it is possible not only to explain the existence of these profound transitions, but also to argue for their position in the parameter-plane of Figure 8. This can be done directly from comparing the mutual action of the different terms of the model.

First we note that the two parameters spanning Figure 8, *k*_growth_ and *k*_shear_, each control one of the two terms that makes up *R*_c_, and that the morphological changes during the adaption dynamics arise through a competition between the growth term, whose function is to increase the number of cells lining the channel lumen, and the shear term, the function of which is to effectively suppress or reverse the same process in the presence of a sufficient concentration of the signaling substance, *m*.

**Figure 10:**
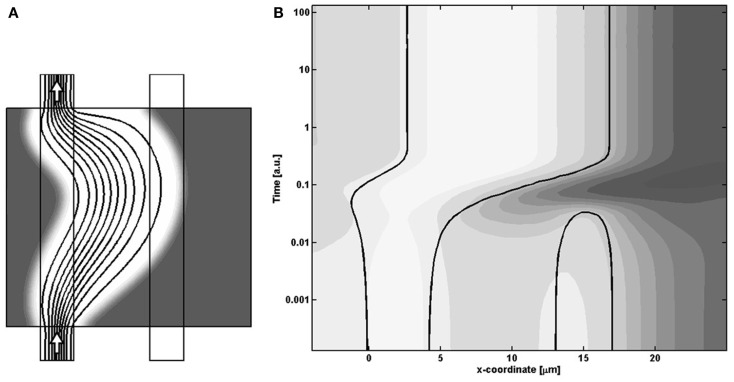
**(A)** Final state and **(B)** center-cut-dynamics, similarly to Figure 7, of the left, poorly performing state of Figure 8. A comparison with the best-performing dynamics in Figure 7 shows that the poor performance arises as the left channel wall does not regenerate, and thereby does not give rise to the overall shift in the position of the channel.

As the right balance between these two terms is crucial for the specific sequence of events leading to the best-performing solution, a proportional shift of both parameters will maintain the terms in balance, i.e., keep the solution and the final morphology inside the central valley. In contrast, “off-diagonal” changes of the parameters will favor one term at the expense of the other, and ultimately either suppress the needed action of the weaker terms and/or enhance the action of the dominating term, such that the adaption dynamics ends up in a less favorable state. This unbalance is illustrated in Figure 10, which is similar to Figure 7 except that the model parameters now correspond to the poorly performing state in the upper left region of Figure 8. By comparing the spatio-temporal adaption dynamics of the two states, it is seen that the poorer state is reached because the cells at the left channel wall fail to spread into the channel lumen when needed, and thereby fail to contribute to the overall channel translation. This defect is anticipated for states in the upper left parameter space region, as this shift both strengthen the shear term and weakens the growth term. Similarly, the opposite shift in parameters leads to another type of failure as seen in Figure 8, which can be explained by a similar failure process, but now originating from a reverse unbalance of the two terms.

## Discussion

The present model is not intended to represent any specific tissue; rather it is aimed describing generic properties present in most tissues in the mammalian body. We consider, as an approximation, structures at two different length scales. On the smallest length scale the tissue behaves like a porous medium. Structures at this length scale are small compared to the rest of the system, (i.e., smaller than the flow channels mimicking larger vessels), hence we consider the tissue with its constituents including capillary sized vessels, as isotropic. Fluid percolates the tissue with a resistance that depends on tissue density. In the model this flow does not take place through well-defined channels but rather as a highly damped flow through the medium. The physiological equivalent of this description is blood flow through a fine capillary meshwork; with tissue density reflecting capillary density. These latter vessels are traditionally considered to be the primary site of exchange between blood and tissue, although exchange does take place in other vessels too (Pittman, 2005). During the adaptation process, tissue density changes dynamically. Eventually though, due to the action of Eq. 6, tissue density outside the large flow channels will increase toward the value 1, as the system settles to a new steady-state.

The larger length scale represents the scale-order of regular flow channels, i.e., vessels larger than capillaries. As the adaptation proceeds, the porous medium is essentially “opened up” by such channels in places that were initially insufficiently perfused. As a consequence, for a given pressure difference across the model region, flow is delivered with increasing efficiency to all parts of the tissue. At some point a balance is reached between the tissue needs and the delivery of the nutritive substance. This causes the production of the substance that signals insufficient flow, to be reduced. In turn, further changes in flow channel topology are suppressed. Eventually as steady-state is reached, the flow-channel is meandering through of tissue. Tissue regions continue to behave like a porous medium but as compared to the situation in Figure 5, the flow-channel now come into sufficiently close contact with all tissue region so as for diffusion to become an efficient mode of mass-transport. As a consequence *a* and *m* become more (though not completely) uniformly distributed (Figure 6).

In the present model we have assumed a certain demand from the tissue (the “tissue need”) for a general nutritive substance, without further specification of which substance(s) the tissue is actually in need of. *In vivo* the tissue must be supplied with a large number of substances including oxygen, food constituents, and hormones, to mention a few. Although we chose to let the nutritive substance represent something that is supplied to the tissue, the removal of waste products such as, e.g., carbon dioxide or lactic acid, is equally important, and could in principle have been used in a similar manner to model a “tissue need”.

The consequence of a certain “tissue need” is that the supply system must adapt dynamically until this need is met. The metabolic turn-over in the tissue utilize the nutritive substance as it is transported through the tissue. Hence its concentration will in general decline with dept inside the tissue, i.e., roughly exponentially with distance from the supplying channels (please also see “*Critique of the model*” below). We assume that for a wide range of flows the cellular metabolism is independent of the flow rate, i.e., the flow is sufficiently large to satisfy the tissue needs, both as regards the supply of nutrients and as regards the removal of waste products. Below a certain local concentration of nutrient however, the requirements of the tissue cannot be met. In that case different cellular responses are elicited *in vivo* including a switch to anaerobic metabolism, cellular hibernation, and ultimately tissue infarction. Hence, cellular metabolism is in itself a function of *a*, but since the abovementioned conditions represent more or less pathological states in the tissue, it seems as if the supply system under normal conditions will adapt so as to reach a capacity where metabolism *per se* does not depend on the flow, i.e., flow is sufficient the majority of time. These properties are expressed in the term for the nutrient uptake rate (Eq. 4). The sigmoid shape of the nutrient uptake rate (Eq. 4) induced by *M_f_(a*; Eq. 5) reflects the dependence of metabolism on the local availability of nutrients. This shape was chosen arbitrarily though, and is not necessarily superior to other similar functions that rise from zero to approach asymptotically a certain maximum value [normalized to the interval (Lorthois and Cassot, 2010)].

Production of the substance signaling insufficient flow follows a similar pattern; as evident from Eq. 8 it is a function of the cellular metabolism, hence its production will essentially cease toward zero as the tissue need is met. On the other hand will *R_m_* increase steeply when metabolism is low due to insufficient concentration of *a*, i.e., at insufficient flow rates. *In vivo* insufficient perfusion elicits a vast array of reactions in the tissue; however as was the case for the lumped description of nutrients (Eq. 4), these reactions was lumped into a single generic signaling function.

A central problem in the mammalian circulation is the regulation of blood vessel diameter. For a physiologically reasonable decline in pressure along a given vessel, it must be able to accommodate the flow needed by the downstream tissues. Similarly on the venous side, a physiologically reasonable decline in pressure must enable adequate drainage of the flow from a given tissue region. Adaptation of vessel size to luminal flow is a well known physiologic mechanism caused by sensitivity to walls-shear stress. In the short run an increase in wall shear stress causes acute, but fully reversible, vasodilatation (Pyke and Tschakovsky, 2005). Sustained changes in wall shear stress on the other hand elicit structural changes (Tulis et al., 1998), which could, at least in part, be due to a persistent influence on the contractile state of the smooth muscle cells of the vascular wall, leading to a “remolding” of the extracellular matrix in the vessel wall (Pistea et al., 2005; VanBavel et al., 2006). Increasing shear stress leads to luminal expansion of the vessel and *vice versa* (Tulis et al., 1998). These changes are evident already after few days but the changes proceeds dynamically until a new equilibrium is established. As a consequence vessels adapt structurally to accommodate the flow needed by the tissue. A similar mechanism operates in the present set equations (Eq. 6). The vessel wall is eroded in response to increased shear, i.e., tissue porosity increases at the edge of the flow channel. Similarly, a reduction in shear causes the surrounding tissue to expand into the channel lumen. Eventually, cell density increases to its equilibrium value (i.e., close to 1) in tissue areas, but is reduced to zero inside the channel lumen, giving rise to the relatively sharp transition between tissue and flow channel seen in Figure 6. Consequently in a given simulation, as shown in Figure 6, flow channels will change position in the tissue and will expand or shrink according to the amount of flow they carry in a given direction which is in turn a function of the amount of tissue being supplied.

### Critique of the model

As all other models the present has its limitations. Some of these will be addressed in the following.

In the present model, flow is diverted to insufficiently perfused regions by a morphological change of the flow channel. The flow channel will change position and meander increasingly until it is sufficiently close to all regions in the tissue. This behavior is mimicking, but is probably not similar to, the same process taking place in the *in vivo* system. In the latter case a change in the main direction of a vessel is likely to take place rather by changing the dimensions of vessels (i.e., by a luminal expansion in all directions) in the vascular segments connected in the direction where more flow is needed. Similarly for vessel segments connected in the direction where less flow is needed the individual vessel will experience luminal shrinkage from all directions. In the end though, the results of the two different processes, simulated or *in vivo*, are comparable, in that the new structure results in a better supply to the tissue. Also the local processes acting on the vascular wall (channel wall) are comparable, but the 2D formulation applied here prevents the formation of branch point since cells can only grow from existing cells and the central part of the flow channel is devoid of cells.

The discussion above points to a central limitation of the model, namely that if the model area is to be extended to cover larger areas it must be able to develop regularly branching vascular network structures. This is not possible in the present 2D formulation; it requires a shift to 3D in which case cells can grow into the channel from the perpendicular direction without obstructing the flow. The latter situation bear a resemblance to intussusceptive angiogenesis *in vivo* (Burri et al., 2004). At present the huge increase in computational load associated with a shift to a full 3D model prevents this approach. Alternatively another initial state could be applied in which for instance the model regions harbors an initial dense network of flow channels which is subsequently pruned, or in which vessel sprouting in some form can take place.

As visualized in Figure 6B, which shows the system in the final configuration, the nutrient concentration, although much more uniformly distributed than in the initial situation just after channel closure, does not become high throughout the system. Ideally, as the system reaches this final state, the general nutrient, *a*, should be present in sufficiently high concentrations throughout the model area to take the nutrient uptake rate into the saturating part of *R_a_*, i.e., resulting from *a* > > *a*_H_. This would result in a more or less complete suppression of the production of *m*. It is however clear from Figure 6B that this is not unequivocally the case in the present model. Looking at the figure there are, in particular in the downstream parts of the model region, areas where the nutrient concentrations is low. This is mainly due to the unrealistically fast reaction kinetics in the model, which are needed for the adaption dynamics to function within the constrained dimensions of the model region. In such a situation of insufficient nutrition concentrations in the downstream parts of the flow channel, one would expect a high concentration of signaling substance, and indeed there exists a high production of *m* close to the outlet channel, but the local concentration of *m* remains low due to its diffusion into the channel lumen and subsequent scavenging and removal by convection. In this way a steady-state can be reached at simultaneously too low concentration of both *a* and *m*.

Finally an unrealistic situation may arise if the concentration of nutrient comes very close to zero. If persisting for a certain period this would *in vivo* correspond to tissue infarction, in which case the tissue would be incapable of producing signaling substances. Clearly, in this case the situation becomes more complex with cell lysis, liberation of cellular constituents etc. Consequently, in the present case we only consider situations where the concentration of *a* is not close to zero.

## Conclusion

In the present work, we have aimed at modeling adaption in a small system to present the working principles of the model. These principles can, with appropriate modifications, be extended to operate on larger structures, and hence potentially be applied to analyze the emergence and adaption of more complex networks. Two main conclusions emerge from the simulations: (1) a simple model of local adaptation to local stimuli can lead to a better tissue supply and (2) the optimal model structure tends to end up close to critical transition zones in the parameter space.

## Conflict of Interest Statement

The authors declare that the research was conducted in the absence of any commercial or financial relationships that could be construed as a potential conflict of interest.

## Footnotes

^1^Actually, for the numerical method, at a given point in time, to further solve the dynamics of the model, it has to evaluate the response from the model in the close vicinity around the current solution, and it is this evaluation, which can reach into un-physical regions of the model, and cause it to fail.

^2^When optimizing such complex systems, it is in practice impossible to guarantee the finding of a global optimum due to the existence of many false optima in the parameter space, and therefore we will consider all such optima as good candidates instead of claiming their global superiority.

^3^This value is close to the measured diffusion coefficient of oxygen in tissue: 1.6 x 10^-9^ m^2^/s (Klitzman et al., 1983).

^4^It should be noted, that the saturated values in the contour plots are not shown in pure black, but rather in dark gray, in order for the black solid streamlines to remain visible even for saturated values.

^5^The following contour figures of model variables will not have scale-bars since the variables by construction are normalized, and further, to emphasize that the model is purely qualitative.
